# From Oversight to Outcome: Addressing the Challenges of Foreign Body Management in Temporal Soft-Tissue Injuries

**DOI:** 10.7759/cureus.70483

**Published:** 2024-09-30

**Authors:** S. Sakthi, V. Venugopalan, G. Satheesh, R. Hemnaath, Narmadha Chandran

**Affiliations:** 1 Oral and Maxillofacial Surgery, Mahalakshmi Multi Specialty Hospital, Arakkonam, IND; 2 Oral and Maxillofacial Surgery, Adhiparasakthi Dental College and Hospital, Melmaruvathur, IND; 3 Oral and Maxillofacial Surgery, Indira Gandhi Institute of Dental Sciences, Puducherry, IND; 4 Oral and Maxillofacial Surgery, Adhiparasakthi Dental College and Hospital, Melmaruvathoor, IND; 5 Oral Medicine and Radiology, Meenakshi Ammal Dental College and Hospital, Chennai, IND

**Keywords:** advanced imaging techniques, foreign body management, learning from mistakes, soft tissue injuries, temporal

## Abstract

This case report details the importance of thorough pre-operative assessment and management of soft tissue degloving injuries to prevent complications. A 55-year-old female experienced pain and bleeding in the right temporal region following a road traffic accident. After the initial suturing, she developed increasing pain, swelling, and purulent discharge, prompting a reassessment. Initially diagnosed with a soft tissue injury and no visible foreign bodies, a CT scan later revealed foreign objects in the temporal region as symptoms persisted. Emergency surgery was performed to remove the foreign bodies, followed by antibiotic treatment and regular postoperative follow-ups. The removal of the foreign bodies led to infection resolution and successful wound healing, with follow-up X-rays confirming no residual materials. This case underscores the necessity of thorough initial assessments, wound irrigation, and meticulous examination to detect foreign bodies in facial injuries, emphasizing the role of accurate surgical techniques and timely interventions in achieving optimal outcomes and preventing complications.

## Introduction

In this case report, we want to establish a maxillofacial surgeon's critical role in conducting a proper assessment while treating soft tissue degloving injuries of temporal skin. Here the patient experienced purulent pus discharge after septic closure of the wound prompting a critical reassessment of the clinical presentation. Subsequent imaging revealed the presence of foreign bodies, including fragments of glass from a car windshield and a glass bangle. This necessitated urgent surgical exploration and meticulous removal of these foreign bodies. 

This case report emphasizes the importance of thorough wound irrigation and site examination by eyes and fingers to detect any foreign bodies, and proper site exposure to prevent any foreign being pouched. Such meticulous assessment not only facilitates early detection of potential complications like infection but also plays a crucial role in reducing the hospital stay duration and the need for subsequent medical visits. By adhering to these principles, surgeons can significantly enhance patient treatment outcomes, ensuring effective management of soft tissue injuries and improving overall clinical care strategies in similar challenging scenarios.

## Case presentation

A 55-year-old female patient presented with a week-long history of pain localized to the right upper quadrant of her face. The pain followed a recent motor vehicle collision involving a truck and a two-wheeler, which occurred approximately 10 days prior. The patient reported no loss of consciousness, emesis, seizures, or otorhinolaryngologic bleeding at the time of the accident. She received initial first aid and wound suturing at a local healthcare facility immediately after the incident.

The patient had no significant past medical or surgical history relevant to her current condition. Upon examination, she was found to be conscious, with stable vital signs, and oriented to time and place. A sutured laceration measuring approximately 7 x 7 cm was noted in the right temporal region. The area was tender to palpation, with evidence of purulent discharge, suggesting a possible infection or foreign body reaction. As part of the examination, the sutures were removed to allow for a comprehensive evaluation of the wound. Despite the tenderness and discharge, no evidence of facial bone fractures was observed on palpation. The patient’s mouth opening was normal, and occlusion was intact.

**Figure 1:**
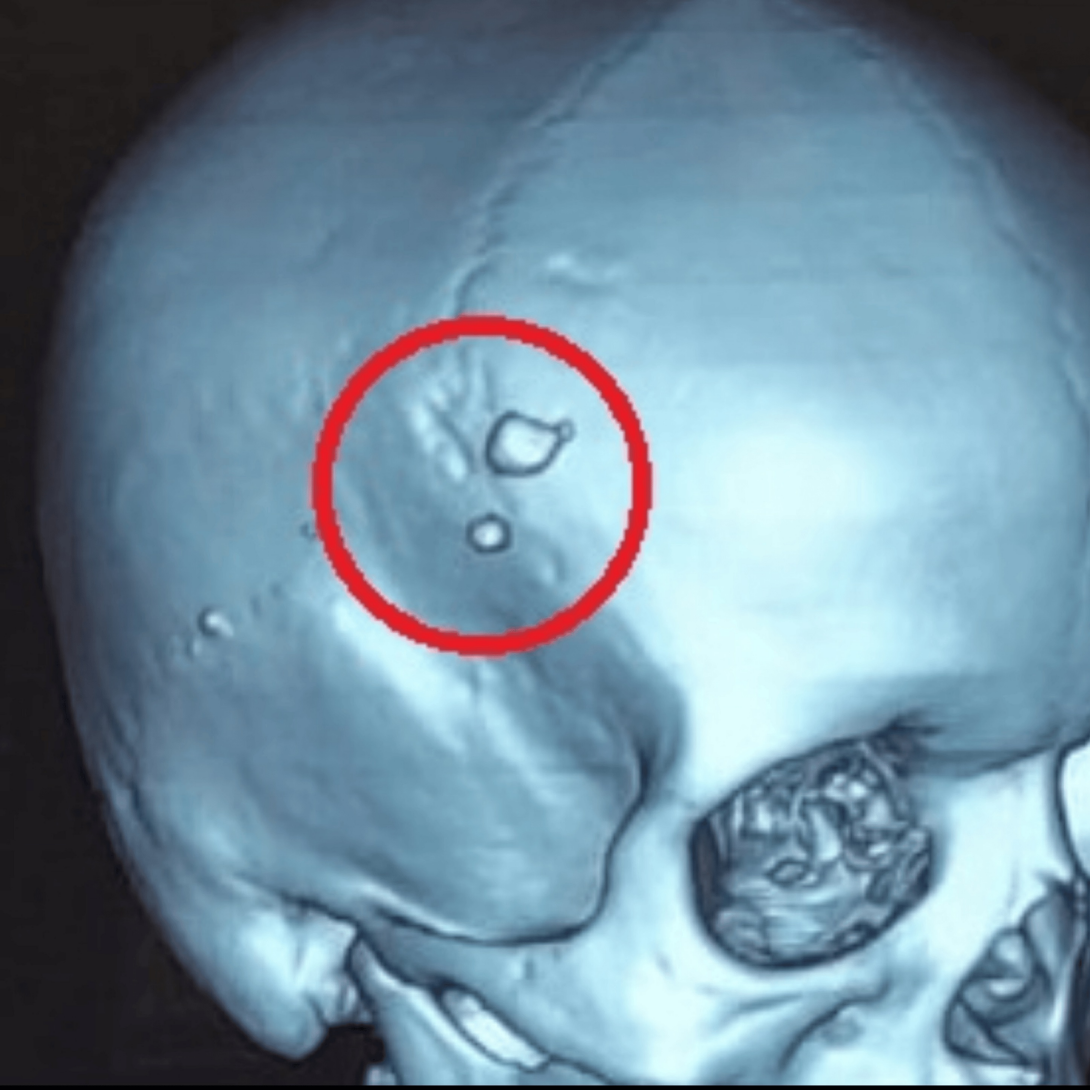
Two radiopaque structures are evident in the 3D CT scan, indicating the presence of foreign bodies at the wound site.

Based on the examination findings, a CT scan of the facial bones was recommended to confirm the presence of any fractures or foreign bodies. The CT scan revealed two radiopaque structures at the wound site, indicating the presence of foreign bodies (Figure 1).

**Figure 2:**
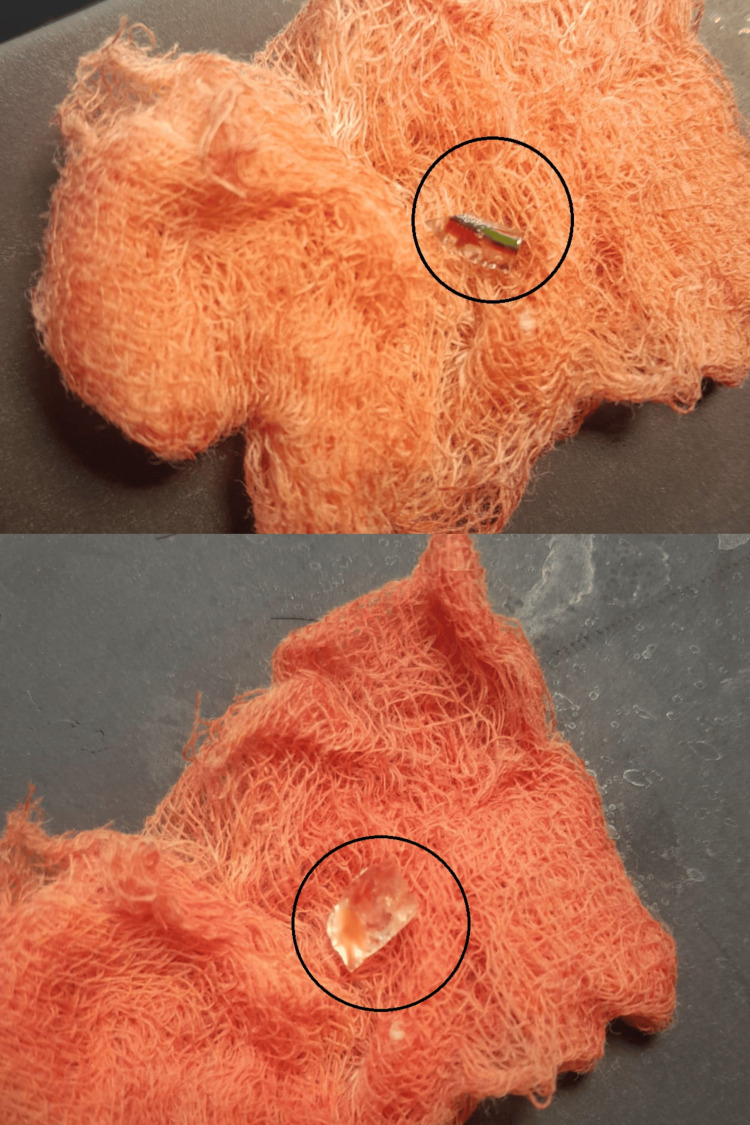
Glass bangle and glass piece from a car windshield retrieved after wound exploration.

An emergency surgical procedure was promptly performed to debride the wound and remove the foreign bodies. The retrieved items included a glass bangle and a piece of glass from a car windshield (Figure 2).

Postoperatively, the patient was administered regular antibiotics. A follow-up X-ray of the skull confirmed the absence of residual foreign materials, and the wound healed without complications.

## Discussion

This case report underscores the critical importance of meticulous assessment and management in treating soft tissue degloving injuries, particularly in the temporal region. It highlights several key aspects: the shortcomings in the initial care provided by the primary doctor, the essential intervention by the oral and maxillofacial surgeon (OMFS), and the lessons learned to prevent similar future occurrences. The primary doctor, responsible for the initial suturing and assessment, failed to perform a comprehensive evaluation of the wound. Inadequate inspection and insufficient wound irrigation led to the retention of foreign bodies, resulting in infection and complications. This oversight underscores the necessity of thorough initial assessments to identify potential foreign bodies and ensure proper wound management. A more detailed clinical and radiological evaluation, including advanced imaging techniques, if needed, could have prevented these complications [1,2].

The role of the Oral and Maxillofacial Surgeon in this case was reassessment. The OMFS played a pivotal role in diagnosing and managing the complications. The OMFS thoroughly examined and utilized a CT scan, which identified foreign bodies, including glass fragments from a windshield and a glass bangle. The emergency surgical procedure performed by the OMFS to remove these foreign bodies was crucial in resolving the infection and promoting proper wound healing. This case highlights the specialized skills and meticulous approach required by OMFS to effectively manage complex facial injuries [3]. We all should learn from mistakes - this case provides valuable insights, emphasizing the importance of thorough wound examination and irrigation to detect and remove foreign bodies.

Key lessons and protocols to prevent similar occurrences

Comprehensive Initial Assessment: A detailed history, physical examination, and appropriate imaging can prevent missed foreign bodies. Conducting a thorough assessment and proper documentation is crucial [1,4].

Advanced Imaging Techniques: Utilizing X-rays as primary imaging, followed by high-resolution CT scans and 3D reconstruction if necessary, aids in the identification of foreign bodies [3].

Enhanced Visualization Tools: Incorporating ultrasound examinations and fluorescence-guided surgery helps in detecting non-radio-opaque foreign bodies [2].

Meticulous Surgical Technique: Adequate wound exposure, intraoperative imaging guidance, and thorough wound irrigation are essential for complete foreign body removal [5].

Surgical Exploration and Removal: Engaging in preoperative planning with a thorough review of imaging data, along with proper wound exposure and irrigation, ensures effective removal of foreign bodies and prevents complications [4].

Postoperative Care and Follow-Up: Immediate postoperative imaging, administration of appropriate antibiotics, and regular follow-ups are necessary to ensure proper healing and avoid recurrence [6,7].

By adhering to these principles and learning from past oversights, clinicians can significantly enhance patient treatment outcomes, ensuring effective management of soft tissue injuries and improving overall clinical care strategies. This case report serves as a reminder of the critical importance of thorough examination, proper surgical techniques, and timely interventions in preventing postoperative complications and achieving optimal patient outcomes [8,9,10].

## Conclusions

In conclusion, this case underscores several critical aspects of managing soft tissue injuries. Thorough initial assessment - a comprehensive examination and appropriate imaging are essential to prevent missed foreign bodies and subsequent complications. Meticulous wound care - effective irrigation, debridement, and postoperative management are crucial for preventing infections and promoting optimal healing. Timely intervention - rapid identification and removal of foreign bodies are vital to prevent prolonged infections and other complications. By applying the lessons learned from this case, clinicians can enhance their approach to managing similar challenging scenarios, thereby improving overall patient care and treatment outcomes.
